# P-2299. Febrile neutropenia in cancer: a retrospective analysis in a dominican tertiary hospital

**DOI:** 10.1093/ofid/ofae631.2452

**Published:** 2025-01-29

**Authors:** Yeison Reyes, Lia Michelle Chaddy Báez, Karla Marie Disla-Pineda, Ruben Calcano, Francisco Guzman-Ricardo, Jose Ledesma

**Affiliations:** Hospital General de la Plaza de la Salud, Santo Domingo, Distrito Nacional, Dominican Republic; Hospital General de la Plaza de la Salud, Santo Domingo, Distrito Nacional, Dominican Republic; Hospital General de la Plaza de la Salud, Santo Domingo, Distrito Nacional, Dominican Republic; SDI, Santo Domingo, Distrito Nacional, Dominican Republic; Hospital General de la Plaza de la Salud, Santo Domingo, Distrito Nacional, Dominican Republic; Hospital General de la Plaza de la Salud, Santo Domingo, Distrito Nacional, Dominican Republic

## Abstract

**Background:**

Febrile neutropenia (FN) poses a significant threat to cancer patients, often leading to severe infections. This study aims to characterize the epidemiology and sources of infection among hospitalized cancer patients experiencing FN at Hospital General de la Plaza De la Salud. By elucidating these factors, we strive to enhance patient care and outcome within our context.

**Figure:**
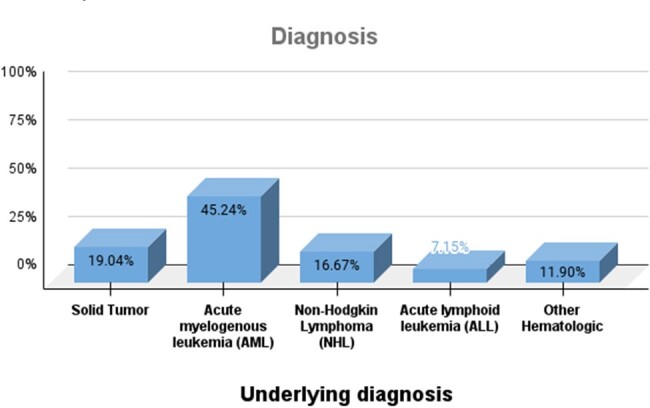

Source: Statistical registry of the authors.

**Methods:**

We conducted a retrospective study at a 289-beds in a tertiary care center in the Dominican Republic from March 2021 to March 2024, assessing 258 hospitalized patients with severe neutropenia. 42 cancer patients met the inclusion criteria (absolute neutrophil count less than 500 cells/µL and fever greater than 38.3°C) and data was collected reviewing the eCharts.

**Figure:**
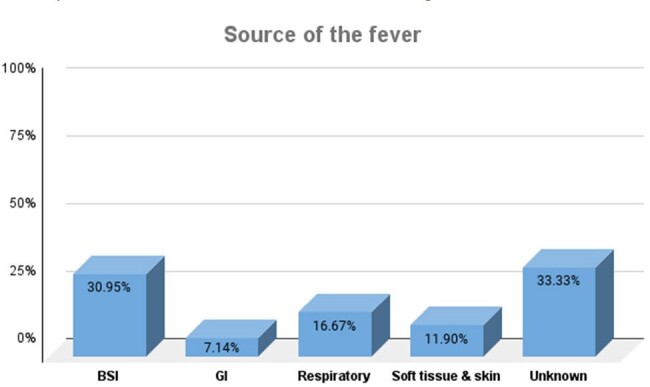

Source: Statistical registry of the authors.

**Results:**

24 patients were male (57.1%), with a median age of 44 (IQR=27). 34 (80.9%) patients had hematologic malignancy, with acute myeloid leukemia being the most common (n=19; 45.2%), followed by non-Hodgkin lymphoma (n=7; 16.7%). Cause of fever was identified for 28 patients, which included bacteremia (13/28; 30.9%), respiratory tract infection (7/28; 16.7%), skin and soft tissue infection (5/28; 11.9%) and others (3/28; 10.7%). An etiological agent was documented in 20 cases, of which the most common was bacterial (15/20;75%); *Pseudomonas aeruginosa* (6/15; 40%), *Escherichia coli* (5/15; 33.3%), and *Klebsiella pneumoniae* (2/15; 20%), 4/20 (20%) were viral; 3/4 due to SARS-COV2 and 1/4 due to dengue, and 1/20 (5%) had *Candida albicans*. 14 (33.3%) patients had fever of unknown origin. 24/42 patients (68%) had a MASCC score < 21. Mortality rate was 30.9% (n=13), of which 12 (92%) had a MASCC score < 21 (p=0.02).

**Figure:**
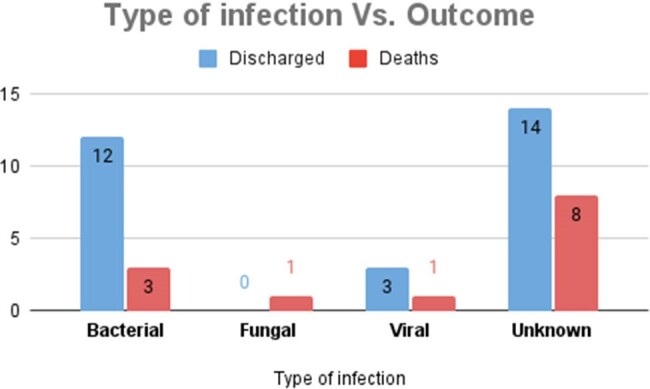

p=0.33.

Source: Statistical registry of the authors.

**Conclusion:**

This study emphasizes how infectious complications greatly impact cancer patients experiencing febrile neutropenia, especially those with hematologic malignancies. Bacterial infections, notably *Pseudomonas aeruginosa and Escherichia coli*, are prevalent, highlighting the urgency of precise diagnostic methods for tailored antimicrobial treatment. The link between low MASCC scores and higher mortality rates focuses the importance of this risk assessment tool in guiding clinical decisions. These findings stress the necessity for improved multidisciplinary strategies to enhance the outlook for this vulnerable patient group.

MASCC risk vs outcome

**Figure:**
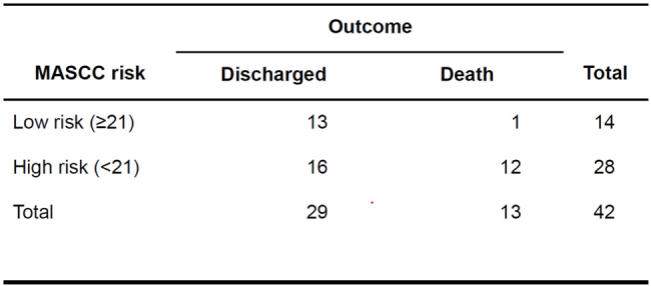

p=0.02

Source: Statistical registry of the authors.

**Disclosures:**

All Authors: No reported disclosures

